# Creating Positive Work Experiences Through Task Self-Redesign

**DOI:** 10.3390/bs9120140

**Published:** 2019-12-05

**Authors:** Severin Hornung, Thomas Höge, Christian Seubert, Jürgen Glaser, Denise M. Rousseau

**Affiliations:** 1 Institute of Psychology, University of Innsbruck, A-6020 Innsbruck, Austria; thomas.hoege@uibk.ac.at (T.H.); christian.seubert@uibk.ac.at (C.S.); juergen.glaser@uibk.ac.at (J.G.); 2 Heinz College and Tepper School of Business, Carnegie Mellon University, Pittsburgh, PA 15213, USA; denise@cmu.edu

**Keywords:** work design, task autonomy, proactive work behavior, job crafting, idiosyncratic deals, positive work experiences, meaning of work, affective commitment, work–home enrichment

## Abstract

Complementing the traditional focus in work design on “top-down” organizational interventions, research into proactive work behavior suggests that “bottom-up” processes, based on the “micro-emancipatory” actions employees engage in, create more rewarding and meaningful work experiences. Based on current theorizing, this study tests a tripartite model of task self-redesign and positive work-related states of meaning, affective commitment, and work–home enrichment. The interactive effects of three modes of task influence are postulated: (a) the active use of existing potentials for task autonomy; (b) job crafting, as unauthorized and self-organized modifications of task features; (c) the individual renegotiation of tasks through idiosyncratic deals (i-deals) with superiors. Survey data from an occupationally heterogeneous convenience sample of N = 279 German-speaking employees were analyzed, using confirmatory factor analysis and moderated linear regression. The regression results confirmed that task i-deals consistently related to positive experiences, whereas autonomy only related to one, and task crafting had no significant main effect. A significant two-way interaction between i-deals and crafting was found only in relation to affective commitment. Supporting the suggested tripartite model, significant (synergistic) three-way interactions explained the additional variance in all three examined outcomes. These results offer some preliminary insights into the interplay of organizationally designed, individually crafted, and interpersonally negotiated work activities. Task autonomy, task-directed job crafting, and task i-deals appear to fulfill complementary roles in the self-directed creation of positive work experiences. Methodological limitations and further research needs are discussed.

## 1. Introduction

Humanistic management theories and positive organizational scholarship emphasize that individuals strive for meaning, fulfillment, and self-actualization in their work [1,2]. This active role of individuals as co-creators of their working situation challenges the traditional emphasis on systematic work analysis and evaluation, the standardization and formalization of job descriptions and essential duties, and broad-based, “top-down” implemented work design interventions. Instead, current concepts of proactive workplace behavior focus on “bottom-up” processes, based on “micro-emancipatory” actions, through which employees create more personally gratifying, meaningful, and enjoyable work experiences—by themselves, in conjunction with other members of the organization, or in their interactions with outsiders, such as customers, clients, or contractors [2,3,4]. Drawing on proactive behavior literature, and integrating theorizing on reciprocal determination between person and environment, social embeddedness, and systemic emergence, this study develops and tests a tripartite model of task self-redesign and positive work-related attitudes or states [5,6,7]. Based on theoretical considerations, the interactive effects of three modes of individual influence over work tasks are suggested and empirically examined: (a) the active use of existing potentials for task autonomy—the degree of job control allowed by the top-down implemented work design; (b) job crafting, as self-organized and unauthorized or unapproved modifications of task features—that is, “bottom-up”, proactive job changes; (c) the individual renegotiation of tasks through idiosyncratic deals (i-deals) with superiors, which covers the middle ground or hybrid processes, i.e., voluntary mutual agreements that are initiated “bottom-up” by individual employees, and “top-down” authorized by employers’ agents (e.g., supervisors, management, HR) [8].

Note that, while all three modes of individual influence at work (task autonomy, job crafting, and idiosyncratic deals) have been subject to research before [2,4,5,8], the main contribution of this study is to investigate them concurrently and with an explicit focus on work tasks, specifically with regard to their interactive effects on working individuals. Based on the understanding that possibilities to exercise control and influence are core to the psychological impact of work and organizational design, these three modes specify zones of personal discretion, organizational indifference, and interpersonal negotiability [8,9,10]. Moreover, they represent different types of top-down, bottom-up, and hybrid processes, through which individual degrees of freedom and associated variability in job features can come about. Drawing on assumptions of person–job fit and positive spillover, the three outcomes of positive work-related experiences chosen for this study were (a) the meaning of work (experienced personal significance and purpose of the work itself); (b) affective organizational commitment (emotional attachment to the employing organization); (c) and work–home enrichment (positive interactions between the professional and private life domains). This set of outcomes is exemplary and eclectic, rather than strictly theory-based or comprehensive. Yet, each indicator has a distinct focus―specifically, the work activity (meaning), the employing organization (commitment), and the relationship between professional and personal life domains (enrichment).

Research hypotheses pertain to autonomy, job crafting, and i-deals as complementary sources of individual influence over work tasks, assumed to jointly contribute to improving the quality of work experiences. Interdependencies among autonomy, job crafting and i-deals are theoretically complex and empirically under-researched [11]. Higher levels of job autonomy have been suggested as both antecedents and outcomes of self-enacted changes [12,13]. Empirically, the negotiation of task i-deals was found to be associated with more positive evaluations of work characteristics (higher complexity and control, and lower stressors), which, in turn, mediated the positive, indirect effects of such personalized work activities on active performance and well-being―in terms of personal initiative and work engagement [8]. Other studies have reported cross-sectional and longitudinal support for a mediating role of job autonomy, between a general measure of i-deals and job satisfaction, as well as between task-focused ideals and supervisor-rated job performance [13,14]. Acknowledging that the reality is likely more complex, the above studies suggest dynamic self-reinforcing processes, based on reciprocal determination between person and environment over time [6,7,15]. Such dynamics are more adequately represented by interactive (moderation) rather than serial (mediation) effects [7]. As research on proactive behavior has established, there are different and potentially parallel, complementary, or alternative pathways for self-enacted improvements in the person―environment fit―as embodied by the influential concept of job crafting [3,5,6]. Job authority and personal discretion were initially seen as determinants of job crafting opportunities, acting as antecedents or moderating factors in conjunction with an individual’s needs for self-actualization [2]. In the job–demands–resources model of job crafting, proactive attempts at increasing one’s zone of autonomy are core to the dimension of increasing structural resources [12,16]. In this quantitative stream, a broad spectrum of antecedents (e.g., demographic factors, individual differences, and job characteristics) and outcomes (e.g., job attitudes, turnover intentions, work engagement, job strain, and different types of work performance) of employees’ job crafting activities have been investigated, partly with longitudinal, diary and interventional study designs [12]. Moreover, preceding research has framed job crafting as psychological self-empowerment, aimed at self-starting improvement in the quality of working life [8,14,16]. Based on these results, job autonomy simultaneously fulfills multiple important functions, such as antecedent, content, outcome, and moderating factors. 

Although frequently discussed on a theory level, the affinities and distinctive features of i-deals and job crafting have not been well established, partly due to the complexity and multi-dimensional nature of both constructs [11]. Focusing on the shared core dimension of work tasks, this study reduces this complexity. The first objective was to demonstrate that task autonomy by work design, unauthorized task crafting, and negotiated task i-deals are distinct [8]. As potentially interacting modes of task-related influence, all three constructs were assumed to make independent as well as joint synergistic contributions to the quality of the individual work experience [7]. Following these considerations, three broader study hypotheses were tested. The first hypothesis (H1) concerns the basic assumption that the three core dimensions of work self-redesign―task autonomy, task crafting, and task i-deals―are empirically distinct constructs. The second hypothesis (H2) predicts independent (i.e., additive) effects of all three constructs and positive work experiences [16], specifically meaning, affective commitment and life enrichment, and that task autonomy (H2a), task crafting (H2b), and task i-deals (H2c) relate to all three included outcomes. Finally, the third hypothesis (H3) goes beyond the second, in proposing that synergistic (i.e., multiplicative or “non-additive”) joint effects are associated with the combined use of control opportunities in the form of task autonomy, task crafting, and task i-deals, such that the three-way interactions of these three core dimensions of work self-redesign explain additional variance in positive work experiences.

## 2. Materials and Methods

### 2.1. Sample

For a preliminary testing of the developed hypotheses, a cross-sectional survey study was used. Survey data were gathered from German-speaking employees in Austria, Germany, Northern Italy, and Switzerland, through personal contacts and networks of students participating in a work psychology course at an Austrian University. For their Bachelor thesis projects, 13 students recruited altogether N = 279 voluntary study participants—all of them employed persons from within their personal circle of relatives, friends, and acquaintances (M = 21.5; SD = 20; Range: 5–88). The students were instructed to contact as many potential participants as possible, with a target range of 20–30 persons each (the number of recruited participants was not a factor in the grading of students). The obtained sample size was at the lower end of the target range, but considered substantive enough for the presented analyses. The students distributed and collected the paper-and-pencil questionnaires provided by the course lecturer (the second author), who was responsible for data entry and quality control. Questionnaires included a cover letter describing the purpose of the research, emphasizing that participation was voluntary, outlining measures taken to ensure anonymity and the confidentiality of data, and providing the name, university, department, and email of the supervising researcher. 

The sample included a broad range of occupations, from education, healthcare, and other person-related services, to administrative and clerical, to manual and technical, as well as managerial and creative professions. More than half were women (59%) and the mean age was M = 35.7 years (SD = 11.6). The majority (> 80%) held open-ended work contracts and were employed full-time with an average of M = 35.5 (SD = 8.7) contractual weekly hours. Actual work hours were estimated at M = 39.0 (SD = 10.0). With a mean of M = 8.9 years, employment tenure was long-term, but included substantial variation (SD = 9.3). More than a third (36%) reported a college or university degree; slightly less (30%) held supervisory roles. Overall, the sample represents a relatively young and highly qualified workforce segment, which was deemed suitable for the purpose of the study.

### 2.2. Measures

Measures were based on established or self-developed German-language scales or previously validated translations. Demographic and employment information was assessed with categorical and numerical items (e.g., sex, age, and education). Altogether 9 multi-item scales were included: (a) 3 core constructs of work self-redesign; (b) 3 additional work characteristics as control variables; (c) 3 aspects of positive work experiences; all used standardized response formats with 4 to 6 options. The complete survey included a number of additional scales (not reported in this study) and required approximately half an hour to complete.

#### 2.2.1. Work Self-Redesign

The 3 components of work self-redesign were each measured with 4 items adapted from previous studies. Task autonomy (α = 0.81; “opportunities for making own decisions in your work”) used the action latitude scale of a validated work-analysis instrument [17]. Task crafting (α = 0.89; “altered the composition of your work tasks”) and task i-deals (α = 0.88; e.g., “negotiated work tasks that suit your personal interests”) were assessed based on measures developed in our own previous research [8,14]. All three used a 5-point response format. 

#### 2.2.2. Work Characteristics

Three additional work characteristics were included as controls. Task complexity (3 items, α = 0.78), task interdependence (4 items, α = 0.81), and task overload (2 items, α = 0.79) were based on established work-analysis instruments [17]. Initiated and received interdependence were combined into a composite measure. Exploratory and confirmatory analyses supported the factor structure. 

#### 2.2.3. Positive Work Experiences

Positive work-related experiences were assessed as the meaning of work, affective commitment, and enriching interactions between work and private life. A 6-item scale on the meaning of work (α = 0.94) was based on a validated meaning and purpose in life questionnaire [18]. Affective commitment (α = 0.88) was assessed with a widely used 5-item scale [19]. Positive spill-over between professional and private life domains was measured with a shortened 4-item scale on work–home enrichment (α = 0.78), drawn from a larger instrument on work–home interactions [20].

## 3. Results

The psychometric properties of the three core variables were established in exploratory (EFA) and confirmatory (CFA) factor analyses [21]. Task autonomy, task i-deals, and task crafting were initially measured with five items each, the psychometrically weakest of which was dropped. Fit indices of the final CFA model with three factors (four items each) fully supported H1. The chi-square discrepancy was non-significant (Χ² = 51.26, df = 51, n.s., Χ²/df = 1.01). The Incremental Fit Index [IFI ≈ 1.00], Tucker Lewis Index [TLI ≈ 1.00], and Comparative Fit Index [CFI ≈ 1.00] all converged towards optimal values of 1.00. The Root Mean Square Error of Approximation [RMSEA = 0.004] was close to zero, with a narrow 90% Confidence Interval [CI = 0.000–0.039] and a non-significant likelihood of a population value above 0.050. In additional analyses, this theoretical measurement model vastly outperformed alternative two-factor and one-factor models. The correlations between the three latent construct factors were: r = 0.47 for autonomy and crafting; r = 0.36 for autonomy and i-deals; r = 0.48 for i-deals and crafting (all *p* < 0.01). These results indicate the distinctiveness and substantial unique variance of core constructs. Latent variables were transformed into manifest scale-level indicators and zero-order correlations with subjective work outcomes were examined. Providing preliminary support for the second hypothesis (H2), all three work self-redesign constructs related significantly positively to all three outcomes. Specifically, raw correlations with meaning, commitment, and enrichment were r = 0.41 / 0.31 / 0.14 (*p* < 0.01 / 0.01 / 0.05) for task autonomy; slightly lower for task crafting, at r = 0.23 / 0.18 / 0.18 (all *p* < 0.01), but strongest for task i-deals, at r = 0.42 / 0.33 / 0.24 (all *p* < 0.01). 

In the subsequent step, the main and interactive effects of the three modes of task flexibility were tested in three separate linear multiple moderated regression models for the three outcomes of positive work experiences (regression results are summarized in Table 1). Controlled and concurrent testing of the main effects of core constructs provides a more rigorous assessment of H2. Regression equations were built-up in several steps. Controls (gender, age, task complexity, interdependence, and overload) were entered first. Next, the main effects of task autonomy, crafting, and i-deals were included. Subsequent steps included the three two-way interactions and one three-way interaction of the three core constructs of work self-redesign. All three models explained significant (*p* < 0.01) variance in the respective outcomes, with R^2^ ranging from 36% for meaning of work, to 21% for affective commitment, and 11% for work–home enrichment, indicating a clear order in the extent to which outcomes were explained by predictors. Surprisingly, task autonomy explained unique variance only in one outcome—meaning of work (β = 0.15, *p* < 0.05), thus lending only partial support to H2a. In contrast, a high level of support was found for task i-deals (H2b), which related to all three positive indicators—specifically, meaning at work (β = 0.25, *p* < 0.01), affective commitment (β = 0.14, *p* < 0.05), and work–home enrichment (β = 0.16, *p* < 0.05). The most inconsistent results were obtained for task crafting (H2c), which, by itself, had no beneficial main effects. Notably, positive three-way interactions (H3) can be observed for all three outcomes, with meaning of work (β = 0.17, *p* < 0.05), affective commitment (β = 0.19, *p* < 0.05), and work–home enrichment (β = 0.20, *p* < 0.05) all suggesting synergistic (non-additive) beneficial consequences of the combination of task autonomy, crafting, and i-deals. This pattern of three-way interaction effects provides full support for Hypothesis 3, that is, evidence of the interplay between the three modes of work self-redesign for an individual’s quality of work experience. Additionally, one two-way interaction between i-deals and crafting was found, such that these two modes jointly boosted affective commitment (β = 0.14, *p* < 0.05). Although specific hypotheses regarding two-way interactions were not part of this study, this finding further highlights the complementarity of i-deals and job crafting as two forms of proactive influence. Graphical plotting and statistical probing moderator effects, through simple slope tests, generally supported the interpretation of synergistic, i.e., mutually reinforcing interactions. No significant two-way interactions occurred with task autonomy, suggesting different mechanisms with regard to task-inherent degrees of freedom. 

Control variables had a number of effects. Women experienced less work–home enrichment (β = −0.13, *p* < 0.05), and older participants higher meaning (β = 0.14, *p* < 0.01). Task interdependence had no effect while complexity was associated with higher meaning and affective commitment (β = 0.19/0.13, *p* < 0.01/0.05); task overload related negatively to meaning (β = −0.15, *p* < 0.01). Regressions were repeated without inclusion of control variables, demonstrating stable results.

## 4. Discussion

Based on conceptual and empirical developments focusing on how individuals proactively create positive work experiences, this study provides an impetus for reviving and extending the perspective of work design and control at work. Task autonomy, task-directed job crafting, and task i-deals played somewhat different, yet complementary roles in the self-directed improvement of working situations. Affective commitment, meaning of work, and work–home enrichment were enhanced by main effects of task i-deals as well as through synergistic three-way interactions of task autonomy, crafting, and i-deals. This suggests the selective complementarity of task i-deals and crafting, a view reinforced by a positive two-way interaction on commitment. Work self-design presents itself as a form of positive organizational behavior, promoting optimal psychological states and work experiences. Results confirm the importance of task autonomy, as well as the synergistic interplay between the negotiation of i-deals and job crafting for employee-oriented outcomes. Most uncertain was the role of task crafting, only stimulating positive responses in conjunction with other forms of control. In this regard, this study deviates from current research that has confirmed the positive effects of job crafting, and using advanced methods, such as diary, multi-level and longitudinal study designs [22,23,24]. This study differs from these investigations by also including task autonomy and idiosyncratic deals, thus separating (i.e., statistically “partializing out”) the effects of these three types of task control, which otherwise may be confounded in the measurement of job crafting. This study shows the potential of a more comprehensive and inclusive control-oriented perspective, integrating different forms of employee influence in their work activity. In this regard, obtained results are promising and warrant future research, but need to be interpreted cautiously in light of the partly exploratory nature of this study and its methodological limitations. 

### 4.1. Limitations

In addition to cross-sectional self-report data, methodological concerns include the sampling procedure and moderate sample size. Employing personal contacts of students, a purposive convenience sampling approach was used. Not unlike “randomly” questioning easily accessible people (e.g., pedestrians), this implies (self-)selection effects—in this case, relating to inclusion in the social network (e.g., friends, relatives) of psychology students at an Austrian university. A comparatively small and occupationally diverse, highly qualified sample was obtained from an economically developed and prosperous region, which needs to be considered when interpreting results. The convenience sampling approach is vastly inferior to the representative stratified sampling of a defined population, thus limiting inferences on generalizability and potentially introducing bias in statistical estimates (e.g., effect sizes). However, the chosen approach also holds advantages, as sample heterogeneity introduces variance in the data on work characteristics and task control, compared to samples drawn from the same organization or occupation. The consistency of three-way interactions for all three outcomes makes it unlikely that these effects are based on capitalization on chance. Nonetheless, research testing their generalizability and potential alternative explanations is needed. Among the latter is the influence of personality traits, which was not considered here. Methodological limitations have to be evaluated relative to the innovativeness of the study. This is the first study to investigate the joint and combined effects of autonomy, job crafting and i-deals, promising to advance research on work design, proactive work behavior, and related streams. Thus, however, results are preliminary and require further investigations testing their external validity, i.e., their replicability in other samples, and robustness with regard to more rigorous testing (e.g., using control variables) and advanced methods (e.g., using multiple data sources and measurement points).

### 4.2. Implications

Practical implications include the proposition that humanistic work design approaches should expand their conventional (rather static) focus on autonomy and participation to take into account alternative forms of control and influence arising from dynamic and reciprocal person–environment and social interactions. Individual negotiation can form the basis for differential components in work design, allowing for interpersonal (across people) and intrapersonal (over time) variability in work tasks. Responsive work design accommodates individualized (person-specific) and individualizing (change-oriented) forms of employee influence—without abandoning its traditional concern for decent collective working conditions [8]. Focusing on meaning, affective commitment, and work–life enrichment, this study emphasizes long-standing humanistic concerns for the positive functions of work for self-actualization, social relatedness, and developmental experiences. The analyzed sample, an occupationally heterogeneous group of mostly highly qualified professionals, seemed suitable for this undertaking. By comparison, on a worldwide scale, this is a rather privileged population. Self-actualization, meaning, and identification may be less relevant when basic conditions of decent work are not met, such as sufficient and secure income, and protection of the physical and psychological safety, health, and wellbeing of the working persons [18]. Efforts to implement the findings presented here resonate with people-oriented humanistic management strategies and should not be employed in instrumental ways to achieve economically advantageous performance increases [25]. While positive outcomes are likely tied to improvements in the overall and long-term quantity and quality of work, these are considered side-effects rather than primary goals of human-oriented work design.

### 4.3. Conclusions

The present study provides a fresh look at different forms of employee control and influence with regard to their work tasks, integrating literature on job autonomy, job crafting, and idiosyncratic deals. The differential and interactive relationships of these three core constructs, with positive employee-oriented outcomes of meaning, identification, and enrichment, offer insights into the relative importance and interplay of organizationally provided, individually enacted, and interpersonally negotiated opportunities to personalize work tasks and on-the-job activities. Improved understanding of these underlying processes is important, particularly when aiming to promote positive employee work experiences, attitudes, and behaviors, to contribute to the overarching humanistic goal of creating healthy, developmental, and productive workplaces.

## Figures and Tables

**Table 1:** Summary of linear multiple moderated regression analyses.

	Meaning of Work (M1)	Affective Commitment (M2)	Work–Life Enrichment (M3)
**Step 1: Control Variables**			
- Gender (0/1 for ♂/♀)	−0.05	−0.04	−0.13 *
- Age (years)	0.14 **	0.03	0.06
- Task Complexity	0.19 **	0.13 *	−0.02
- Task Interdependence	−0.06	−0.05	−0.01
- Task Overload	−0.15 **	−0.10	−0.04
**Step 2: Core Constructs**			
- Task Autonomy (TAU)	0.15 *	0.13	−0.04
- Task Crafting (TCR)	−0.05	0.01	0.05
- Task I-deals (TID)	0.25 **	0.14 *	0.16 *
**Step 3: Interaction Effects**			
- TAUxTCR	0.04	−0.04	0.07
- TAUxTID	−0.10	0.04	0.00
- TCRxTID	0.09	0.14 *	0.07
- TAUxTCRxTID	0.17 *	0.19 *	0.20 *
**Model Summary**			
Multiple R^2^ (adjusted R^2^)	0.36 (0.33)	0.21 (0.18)	0.11 (0.07)
F (12; 260)	11.97 **	5.83 **	2.70 **

Note: N = 279; ** *p* < 0.01, * *p* < 0.05; standardized regression coefficients (ß-weights).
